# Doctors in Parliament

**Published:** 1918-10-12

**Authors:** 


### The Hospital

The Workers' Newspaper of Administrative Medicine and Institutional Life, Administration, National Insurance and Health.

No. 1688, Vol. LXV. SATURDAY, OCTOBER 12, 1918. PRICE TWOPENCE

### DOCTORS IN PARLIAMENT.

If we may judge from the comments of our lay contemporaries the proposal that the medical profession should be adequately represented in the House of Commons commands universal approval. Everywhere it is recognised that the legislation of the immediate future is to be largely concerned with measures which relate to the health and physical well-being of the people, and that efficient guidance in such enterprises can only be obtained from medical knowledge and counsel. The most obvious way to obtain help of this order would seem to be the election of experienced and competent doctors as members of Parliament. It may be said, indeed it is said, that legislators and administrators, when they desire it, can always obtain an expression of medical opinion from various professional organisations or from the professional officers who form part of the administrative machine. No doubt this has been the practice hitherto, but it cannot be said that such a method has been entirely satisfactory to the profession. What official doctors announce to their political chiefs may have a note of authority, but no one is in a position to say that it really represents the deliberate judgment of the medical profession. And a similar comment must be made on advice tendered by such bodies as the Councils of the Royal Colleges, for these are narrow organisations over which, not only the profession generally, but even their own members have no authority or control. This arrangement may perhaps be advisable or necessary for certain purposes, but it is fatal to the claim that such councils hold a representative voice. In short there does not exist any organisation which is competent to act as an organ of professional opinion in the widest sense of this word, for, while the British Medical Association may indeed claim a large membership, many members of the profession stand outside its ranks and not a few of its own adherents render it a merely nominal support. Further, in all discussions between these professional bodies on the one hand and Government officials and legislators on the other there is an element of secrecy, and the avoidance therefore of any opportunity for general criticism and discussion. If the profession is represented it is represented behind its back, and no one is quite sure what word has been spoken.

Now, obviously, all these evils would be avoided were a number of members of the House of Commons duly accredited to speak authoritatively in the name of their profession. The public would then have a guarantee that the real voice of the profession on questions relating to health legislation would be spoken, and the profession itself would be content with the knowledge that its mind had been openly and unhesitatingly declared. Nor can the value of the personal factor be ignored. Deputations, associations, representations, petitions, and the like, have a quality which even when impressive is but brief, and the experienced and chastened member of Parliament is apt to conclude that there is more sound than substance in their operations. But a fellow-member, known and respected by his colleagues, and speaking with the emphasis of personal knowledge and conviction, is an advocate of a very different order, and, moreover, his intervention can take place at the appropriate moment, that is when legislative projects are still in the fluid stage and are still therefore susceptible to modification and amendment. If a body of medical members capable of helping to frame legislation in accordance with scientific demands and popular need, and speaking with the undoubted support of their colleagues, could be secured for the House of Commons the advantage to the community would be a considerable one, and at the same time the profession would obtain a fair opportunity for the protection of its legitimate interests and for the argument that these interests and those of the community are in harmony, and not in conflict, the one with the other.

But while all is smooth sailing so long as the discussion is concerned merely with what is desirable, difficulties immediately appear when the agreed aim has to be translated into practice. First, as already pointed out, there is no organ of professional opinion which may claim to nominate representative medical men as members of the House of Commons, and even if such a body existed it would have no legal capacity to act in this direction. The notion that doctors as such might form one or more special constituencies with particular representation in Parliament can only be regarded as an impracticable dream. The whole trend of democratic activity is against a proposal which would immediately arouse a kindred claim from many other sectional and equally plausible interests. Parliament, it may be taken as certain, will take no special measures to secure the presence of doctors in the House, and if doctors are to get there they must help themselves. They must, even as others, take their share in the rough-and-tumble of politics, and while perchance they may find an attenuated form of this discipline in the relatively calm atmosphere of the university constituencies, they must at the best adhere to a political party and be either buff or blue. If the profession can find suitable men, a general character may be lent to their voices by the knowledge that money for the enterprise is provided by the profession, and that in some degree the elected members speak with the support of a recognised professional organisation. But it is folly to imagine that either Parliament or the public is going to organise some special non-political machinery in order to bring doctors into the House of Commons. If they can get there, well and good. But there will be no royal road either for them or for others.

